# High-throughput proteogenomics of *Ruegeria pomeroyi*: seeding a better genomic annotation for the whole marine *Roseobacter *clade

**DOI:** 10.1186/1471-2164-13-73

**Published:** 2012-02-15

**Authors:** Joseph A Christie-Oleza, Guylaine Miotello, Jean Armengaud

**Affiliations:** 1 CEA, DSV, IBEB, Lab Biochim System Perturb, Bagnols-sur-Cèze, F-30207, France

## Abstract

**Background:**

The structural and functional annotation of genomes is now heavily based on data obtained using automated pipeline systems. The key for an accurate structural annotation consists of blending similarities between closely related genomes with biochemical evidence of the genome interpretation. In this work we applied high-throughput proteogenomics to *Ruegeria pomeroyi*, a member of the *Roseobacter *clade, an abundant group of marine bacteria, as a seed for the annotation of the whole clade.

**Results:**

A large dataset of peptides from *R. pomeroyi *was obtained after searching over 1.1 million MS/MS spectra against a six-frame translated genome database. We identified 2006 polypeptides, of which thirty-four were encoded by open reading frames (ORFs) that had not previously been annotated. From the pool of 'one-hit-wonders', *i.e*. those ORFs specified by only one peptide detected by tandem mass spectrometry, we could confirm the probable existence of five additional new genes after proving that the corresponding RNAs were transcribed. We also identified the most-N-terminal peptide of 486 polypeptides, of which sixty-four had originally been wrongly annotated.

**Conclusions:**

By extending these re-annotations to the other thirty-six *Roseobacter *isolates sequenced to date (twenty different genera), we propose the correction of the assigned start codons of 1082 homologous genes in the clade. In addition, we also report the presence of novel genes within operons encoding determinants of the important tricarboxylic acid cycle, a feature that seems to be characteristic of some *Roseobacter *genomes. The detection of their corresponding products in large amounts raises the question of their function. Their discoveries point to a possible theory for protein evolution that will rely on high expression of orphans in bacteria: their putative poor efficiency could be counterbalanced by a higher level of expression. Our proteogenomic analysis will increase the reliability of the future annotation of marine bacterial genomes.

## Background

The first complete bacterial genome to be sequenced was that of *Haemophilus influenza *[1]. Seventeen years later, techniques for sequence determination and automated annotation tools have improved dramatically [2]. Genome sequences are now considered to be highly redundant and thus accurate when fully assembled. However, genome annotation is still far from being perfect, either in terms of structure (precise location of gene starts, regulatory sequences, etc.) or in terms of functional assignments [3,4]. An *in-silico *genome analysis estimated almost 60% erroneous start codon prediction in some prokaryotic genomes [5]. The genomes of almost 1600 living cellular organisms from the three domains of life have been sequenced and annotated to date: 1460 bacteria, 105 archaea, and forty eukarya (2011/05/21 update). The annotation of subsequent thousands of genomes expected to be released within the coming months (the annotation of 4906 microbial genomes is currently in progress) will rely, in almost all cases, on automated annotation pipelines and will be deposited as such in repository databases with no manual verification [6].

New strategies have been proposed to better annotate genomes with the integration of experimental data collected at the transcriptome or proteome levels (for a review, see: [7]). The expressed genome can give a reliable refinement of genome annotation and can be further extended to other related genomes by comparative genomics. In this way, massive transcriptome sequencing (RNA-seq) has been carried out for *Caenorhabditis elegans *[8] and *Vitis vinifera *[9], producing a large list of novel, transcribed sequences and alternative splicing information. However, many RNAs are non-coding and, therefore, coding RNAs that exhibit low similarities with other sequences should be further confirmed. Hence, a more direct analysis of proteins is recommended. Recent improvements in mass spectrometry have allowed high-throughput protein analysis by shotgun nanoLC-MS/MS, which can generate useful information on thousands of proteins [10,11]. The integration of proteomic data into a nucleotide database translated in the six reading frames, in order to improve genome annotation, was first proposed by Yates and co-workers [12] and has subsequently been applied at a large genomic scale by many research groups [3]. The resulting information is used to identify novel genes that were missed in the first annotation and to correct annotation mistakes [7]. The mapping of mass spectrometry-certified peptides onto the nucleotide sequence has been applied at the primary annotation phase for at least three microorganisms: *Mycoplasma mobile *[13], *Deinococcus deserti *[14], and *Thermococcus gammatolerans *[15]. Integrating both transcriptomic and proteomic complementary approaches has already been carried out for *Pristionchus pacificus *[16] and *Geobacter sulfurreducens *[17]. The main drawback of both approaches is that only a fraction of the transcriptome or the proteome can generally be observed under standard laboratory culture conditions for generalist lifestyle organisms, *i.e*. those with large genomes [18].

On the basis of evolutionary constraints, re-annotations obtained by proteogenomics for one organism can be extended to all orthologous genes present in all phylogenetically related species. This approach, first proposed by Gallien *et al. *[19] for the *Mycobacterium *genus, was tentatively called ortho-proteogenomics. A similar study has also been performed on *Yersinia pestis KIM *and extended, respectively, to the twenty-one closest *Yersinia *species sequenced [20]. The integration of proteogenomic studies carried out on closely related species addresses several notorious problems encountered with mass spectrometry approaches. Such is the case of the 'one-hit-wonders', proteins detected with only one MS/MS identified peptide which need to be evaluated with caution.

The *Roseobacter *clade is a group of bacteria that can represent up to 20% of bacterioplankton communities in coastal environments and more than 10% in the open ocean [21]. The first *Roseobacter *genome to be sequenced was that of *Ruegeria pomeroyi *DSS-3. This genome consists of a 4.11 Mb chromosome and a 0.49 Mb megaplasmid, named pDSS-3 [22]. A high number of *Roseobacter *isolates have since been sequenced and many more are in progress in order to explore this important clade and to understand how ocean ecosystems function.

In the present study, we propose a re-annotation of the *R. pomeroyi *genome using experimental data obtained from a large proteomic survey recorded for this micro-organism. This ortho-proteogenomic study is the first to propose the extension of proteogenomic corrections to a large bacterial clade. Moreover, we have detected highly abundant orphan proteins and discuss their significance.

## Results

### Proteogenomic strategy for the analysis of *R. pomeroyi*

**Figure 1:**
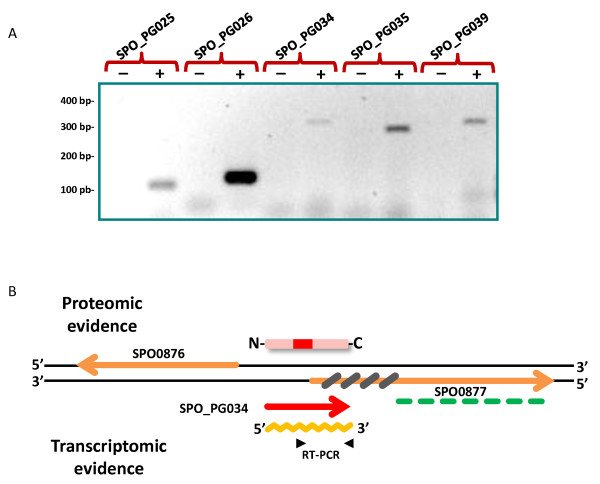
**RT-PCR amplification for 'one-hit wonder' validation**. A schematic view of the genomic region of target SPO_PG034 is shown as an example. The pink square represents the putative protein sequence highlighting in red the unique peptide detected by MS/MS. SPO0877 is shown with its conserved BLAST region (broken green line) and plausible non-coding area (grey crossed). In yellow is the mRNA produced from SPO_PG034 which was amplified with by RT-PCR using specific primers. The 3% agarose gel stained with ethidium bromide shows the five "one-hit wonder" targets from which RT-PCR amplification was obtained (lane "+"). Lanes marked with "-" represent negative controls by testing PCR amplification on RNA extractions to ensure total DNA elimination.

An extensive analysis of the proteome of *R. pomeroyi*, cultivated in various conditions, resulted in a large dataset of MS/MS spectra (1,117,372). To assign these spectra to peptide sequences, we created a database comprising all translated stop-to-stop amino acid sequences of more than forty residues, taking into account the six-frame translation of the genome of *R. pomeroyi*. Thus, this database comprises all the coding domain sequences (CDS) of the bacterium mixed with a large number of aberrant polypeptide sequences. All of the MS/MS spectra were searched against this database using the Mascot engine, resulting in the identification of a restricted set of 4425 probable ORFs. A second search against this restricted database led to the assignment of 594 902 spectra corresponding to 22 805 non-redundant tryptic peptides (Additional file 1). These peptides validated the presence in the cells of 2006 polypeptides detected with at least two peptides (Additional file 2). When comparing these mass spectrometry-certified ORFs with the 4252 previously annotated CDSs [22], we found that thirty-four ORFs were missing. Table 1 lists the corresponding proteins with their probable start and stop positions, as well as their putative function. Remarkably, most of these are unknown. We also took into consideration those novel ORFs detected with only one highly confident peptide (*p*-value under 3.2e-5) and showing no overlap with other proteomic-detected genes. Applying these criteria resulted in the selection of seven targets. To give higher confidence to the validation of these 'one-hit-wonders', we checked whether these genes were transcribed. We extracted total RNA from bacteria grown in the physiological conditions in which each polypeptide had been most abundantly detected during the proteomic survey. We performed RT-PCR amplification for five of these targets (**Panel A **of Figure 1; Table 1). Supplementary Additional file 4 shows the 181 non-redundant peptides assigned to the thirty-nine novel CDSs to be subsequently considered. The culture conditions and proteome fraction in which each peptide was best detected is also indicated. Regarding these CDSs, we propose a novel nomenclature to indicate that their existence was experimentally verified by proteogenomics. It consists of the introduction of the two letters 'PG', standing for ProteoGenomics, and a number beside the SPO nomenclature for annotated CDSs encoded on the bacterial chromosome and SPOA for those coded by the pDSS-3 megaplasmid (Table 1). We also identified ten ORFs with peptides located further upstream than their annotated N-termini (Additional file 5). This clearly indicated a wrongly identified translation start codon for the corresponding gene. We confirmed these corrections by Blast analysis. In addition, we listed in a second query the semi-tryptic peptides that could correspond to the most-N-terminal peptide, as detailed elsewhere [23].

**Table 1:** List of novel genes found in the genome of *R.pomeroyi *detected by proteogenomics.

	Target ^a^	Plausible CDS start	Stop	Length (aa)	Peptides assigned	CDS Proteomic coverage	Function/Presence in other Roseobacter stains ^b^
**Non-annotated CDS**	SPOA_PG001	300016	300507	164	10	73%	Unknown/observed (9e-51)
	SPO_PG002	3171305	3170874	144	9	69%	Unknown/unique
	SPO_PG003	1412876	1413418	181	7	73%	Unknown/observed (5e-23)
	SPOA_PG004	87032	87709	226	6	27%	Unknown/unique
	SPO_PG005	358784	358125	220	6	45%	Esterase-lipase/observed (5e-45)
	SPO_PG006	360911	360405	169	5	54%	Unknown/unique
	SPO_PG007	1483195	1482533	221	5	48%	Unknown/unique
	SPO_PG008	1431167	1431595	143	5	45%	Unknown/observed (3e-56)
	SPO_PG009	501740	502171	144	5	42%	Unknown/unique
	SPO_PG010	2353576	2353965	130	4	42%	Unknown/observed (1e-38)
	SPO_PG011	1374589	1374299	97	3	61%	Unknown/conserved (1e-43)
	SPO_PG012	3703461	3702955	169	3	22%	Unknown/unique
	SPO_PG013	649156	649749	198	3	23%	Unknown/unique
	SPO_PG014	2482691	2482317	125	3	20%	Unknown/unique
	SPO_PG015	3657397	3656924	158	3	19%	Unknown/observed (6e-50)
	SPO_PG016	373055	373333	93	2	41%	Unknown/unique
	SPO_PG017	1092236	1092592	119	2	34%	Unknown/unique
	SPO_PG018	495167	495529	121	2	22%	Unknown/observed (4e-44)
	SPO_PG019	1418666	1419187	174	2	10%	Signal transduction/conserved (1e-69)
	SPO_PG020	2807747	2807223	175	2	19%	Polyketide cyclase/unique
	SPO_PG021	1289473	1289829	119	2	28%	Unknown/unique
	SPO_PG022	1151078	1151632	185	2	18%	Unknown/unique
	SPO_PG023	1400166	1399696	157	2	24%	Unknown/unique
	SPO_PG024	2628409	2629668	420	2	9%	RNA helicase/conserved (1e-175)
	**SPO_PG025**	1322016	1322357	114	1	7%	Transcriptional regulator/unique
	**SPO_PG026**	3883013	3882531	161	1	7%	Unknown/unique

**Wrong CDS**	SPO_PG027	501090	501710	207	21	77%	Unknown/unique
	SPO_PG028	2429044	2427941	368	20	63%	Unknown/conserved (5e-92)
	SPO_PG029	3124885	3123728	386	11	36%	Sporulation related/conserved (6e-92)
	SPO_PG030	1738173	1736680	498	7	24%	Unknown/conserved (1e-175)
	SPO_PG031	2905673	2906335	221	6	37%	Unknown/unique
	SPO_PG032	3751605	3751147	153	6	42%	Unknown/conserved (5e-42)
	SPO_PG033	2357076	2357507	144	2	18%	Excinuclease/observed (4e-35)
	**SPO_PG034**	934724	935068	115	1	17%	Unknown/Observed (1e-27)
	**SPO_PG035**	2751483	2750281	401	1	4%	Unknown/Conserved (1e-162)

**Seq. error**	SPO_PG036	562052	560282	590	3	9%	ABC transporter/conserved (0.0)
	SPO_PG037	3188876	3188459	139	3	27%	Heat shock protein/observed (3e-55)
	SPO_PG038	2152217	2151179	346	2	10%	Aminotransferase/conserved (1e-168)
	**SPO_PG039**	3515528	3515111	139	1	17%	Stress protein/unique (conserved in *Bacillus*)

^a ^Targets in bold represent those "one-hit-wonders" validated by RT-PCR.

^b ^*Observed *indicates presence of a similar gene in less than 5 other *Roseobacter *strains whereas *Conserved *means presence in over 20 of the 36 strains searched. E-value for BLAST analysis with its nearest homologue is indicated in brackets.

### Novel CDSs found in, *a priori*, non-coding genomic regions

As shown in Table 1, the SPO_PG001-026 CDSs were found to be encoded in genomic regions encompassing no previously annotated genes. Among them, sixteen have no known homologues in any other sequenced *Roseobacter *strains, while seven are homologous to proteins found in a limited number of *Roseobacter *strains. Generally, these genes encode small polypeptides (with a mean length of 166 amino acids) whereas the mean length of polypeptides encoded in the genome is greater than 320 amino acids. Curiously, the well conserved RNA helicase, SPO_PG024, was not previously annotated, due to the report of a putative translational frameshift in the sequence [22]. We definitively discarded this possibility due to the detection of a peptide situated from amino acid position 432 to 454 in the stop-to-stop amino acid sequence, with the hypothetical frameshift in position 451. The sequence between this position and the end of SPO_PG024 is badly conserved among the different *Roseobacter *members, being a possible hotspot for the accumulation of mutations and the reason why a putative frameshift was at first predicted.

### Novel CDSs indicating badly-annotated genes

**Figure 2:**
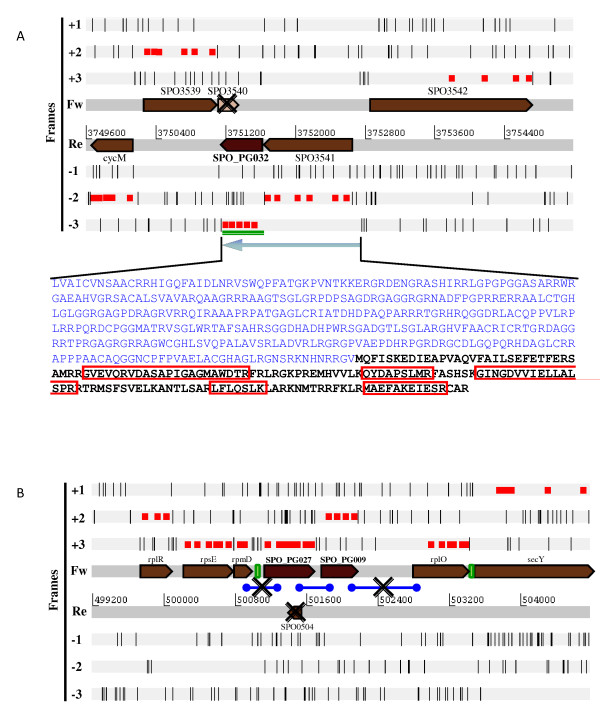
**Chromosome region view of three novel genes detected by proteogenomics**. Loci of targets SPO_PG032 (**Panel A**) and targets SPO_PG027 and SPO_PG009 (**Panel B**) are represented. The six reading frames are shown with all coded stop codons represented by black dashes. Coloured in red are the nucleic sequences specifying the peptides detected by MS/MS. **Panel A**: The green line represents the area of the stop-to-stop sequence that shows homology to other proteins by means of a PSI-BLAST. The amino acid sequence in bold black represents the plausible sequence of SPO_PG032. Highlighted in red squares are the peptides detected by MS/MS. **Panel B**: Blue lines represent RT-PCR amplification attempts. Green rectangles show identified transcriptional terminators.

SPO_PG027-035 CDSs were found to overlap with previously annotated CDSs which had not been detected during the proteomic analysis. Moreover, the previously annotated CDSs did not exhibit any similarities with other bacterial proteins, as revealed by means of a PSI-BLAST search. Seven of the new proposed genes whose products were detected by tandem mass spectrometry showed high similarities to proteins encoded in other related members of the *Roseobacter *clade (Table 1). **Panel A **of Figure 2 represents the chromosome region view of the target SPO_PG032. It shows how the tryptic peptides identified allow a better definition of each CDS on the genome, with no overlap between them. This figure also illustrates how the detection of the novel ORF, SPO_PG032, is in total discordance with the possible existence of SPO3540.

Remarkably, twenty-one distinct peptides cover 77% of the full SPO_PG027 polypeptide sequence. This protein was detected abundantly in the experimental conditions tested in the present study, with 752 spectral counts assigned to it (Additional file 4). Its sequence does not exhibit any similarities to other known proteins in any other sequenced organism. This specific gene, found in the *R. pomeroyi *genome, probably plays an important role in this bacterium as it was systematically detected in all of the biological samples analysed. **Panel B **of Figure 2 shows that this novel gene is found next to SPO_PG009, another re-annotated CDS, and both are located between two conserved ribosomal protein operons. Curiously, this intergenic region of 1.8 kb in *R. pomeroyi *presents a high variability among the closest sequenced *Roseobacter *members. This genomic area presented a variable size (0.1-1.5 kb) and sequence, with no inter-species similarity. The existence of the previously annotated SPO0504 gene is undoubtedly false. It was assigned to the other DNA strand and the corresponding short polypeptide product (forty-one residues) did not show any similar protein by PSI-BLAST. SPO_PG028 is also highly detected by MS/MS as 1395 spectra were assigned to this polypeptide (Additional file 4). In contrast to SPO_PG027, SPO_PG028 is highly conserved in other members of the *Roseobacter *clade. Curiously, SPO_PG028 was missed, favouring the annotated SPO2290 CDS, which gives no sequence homologue by means of PSI-BLAST.

Interestingly, the stop-to-stop ORF comprising SPO_PG031 overlapped the stop-to-stop ORF comprising an already annotated gene (SPO2724) detected during the proteomic survey. SPO2724 was validated with twenty non-redundant peptides (Additional file 2) but showed no similarities to other proteins by means of PSI-BLAST. The first validated peptide of SPO2724 was identified 901 nucleotides downstream of the initially annotated start codon. This indicates that this gene could be shorter, resulting in no overlap with SPO_PG031. A similar case is that of SPO_PG034 (one-hit wonder example represented in Figure 1, **Panel B**). The C-terminal region of this newly detected gene overlaps the N-terminal region of the stop-to-stop SPO0877 ORF. A PSI-BLAST search with the annotated sequence of SPO0877 clearly indicates, by comparison with its closest homologues, that this conserved esterase lipase in *R. pomeroyi *should be 127 amino acids shorter. In such a case, the start codon of SPO0877 would be in position 935 284 on the chromosome, being consistent with the existence of SPO_PG034.

### Detection of sequencing errors

**Figure 3:**
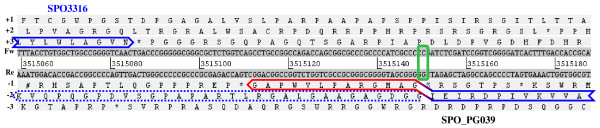
**Locus of a sequencing error detected in the SPO_PG039 sequence**. The reading frame of this novel detected gene shifts from frame -2 (highlighted in blue) to frame -1 (in red) due to an erroneously annotated extra nucleotide (highlighted in green).

SPO_PG036-038 CDSs are highly conserved in other bacteria and all have putative assigned functions (Table 1). Like SPO_PG024, these genes were not considered at the primary stage of annotation (Moran 2004) as they all presented a putative frameshift in their sequence. For the targets SPO_PG036 and SPO_PG037, we detected two peptides located in one reading frame, as well as a third peptide that could belong to the same polypeptide but in another reading frame (Additional file 4). We double-checked the sequence of the nucleotide region where the plausible frameshift could occur and demonstrated a sequencing error in all four targets. In all cases we found an extra nucleotide in the sequence, which should be removed (an extra G at position 561 870, a T at position 3 188 646, a G at position 2 151 427, and a G at position 3 515 150, respectively, for each of the targets). These extra nucleotides led to changes in the reading-frame. The corrected protein sequences of these four targets are indicated in Additional file 4. They result in full-length proteins with higher similarities with their respective homologues. SPO_PG039 is an interesting case revealed by the detection of a unique peptide. The corresponding polypeptide sequence does not share similarities with any protein present in other sequenced *Roseobacter *members, but has some similarity with a conserved stress response protein present in the genus *Bacillus*. As shown in Figure 3, the corresponding stop-to-stop ORF in the genome of *R. pomeroyi *overlapped the highly conserved annotated gene, SPO3316. The single peptide detected for this target, and the region exhibiting similarities to the stress response protein of *Bacillus*, both reside at the N-terminus of the ORF, in between the annotated genes, SPO3316 and SPO3317. When the extra G was removed, the reading-frame of SPO_PG039 was corrected, ending at a stop codon (position 3 515 110) before overlapping SPO3316 (Figure 3).

### Polypeptide N-terminus validation and correction

To confirm or correct the annotation of translational start codons of the CDSs, we first searched for strictly tryptic peptides located upstream of those previously annotated in the stop-to-stop ORF sequence. In addition, we searched for semi-tryptic peptides to identify possible authentic N-terminal peptides.

**Figure 4:**
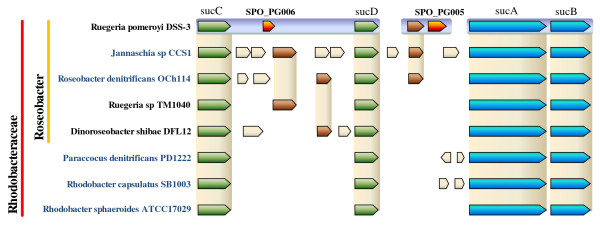
**Genome conservation of operons *sucAB *and *sucCD *between *R. pomeroyi *and its seven closest *Rhodobacteraceae *members**. The comparison was carried out by a BLAST analysis. In green and blue are those conserved genes that make up the operons. In orange are the novel genes reported in this work. Brown genes represent those genes that share identity with other genes in *Roseobacter *members.

The search for peptides located further upstream than the previously annotated start codons [22] led to the discovery of ten wrongly annotated CDSs. These are listed in Additional file 5. The ten resulting N-terminal extensions did not overlap other genes. Moreover, higher similarities were found by PSI-BLAST with homologous genes annotated in other related species. Of note, one of these corrected start codons is that of the conserved *sucB *gene, SPO_0343, which is just downstream of *sucA *(SPO_0344), as shown in Figure 4. Another interesting case is that of SPO_1905, which shows high sequence similarities with its homologues found in the *Roseobacter *clade. However, the first residues were found to be very dissimilar in comparison with its closest homologues. We sequenced the locus and found a sequence error with an extra G between positions 2 029 022 and 2 029 023. This would be the reason why a shorter sequence was at first assigned to this gene. The corrected N-terminus of the polypeptide sequence is now similar to its closest homologues.

The search for semi-tryptic peptides led to the discovery of the N-terminal peptides of 486 proteins (Additional file 6). All of these were confirmed by PSI-BLAST comparison with their counterparts in other species and by manual inspection: 422 peptides confirmed already well-annotated start codons, sixty-two peptides corresponded to genes with an erroneously identified start codon, and two were the N-terminal peptides of the newly annotated genes, SPOA_PG001 and SPO_PG024, identified in this work (Table 1). These data reveal that almost 13% of the CDSs annotated at the primary stage [22] could have a wrongly identified start codon. As expected, the ratio of initiator codons is usual for bacteria: ATG (94%), GTG (5%) and TTG (1%). Over 87% (54/62) of the wrongly annotated CDSs were shorter than previously annotated. This was as expected and is probably due to the GLIMMER annotation system used, which tends to privilege annotation producing the longest possible sequence.

### Seeding proteogenomic annotation for the whole *Roseobacter *clade

An ortho-proteogenomic analysis was carried out in order to extend the genomic re-annotations proposed for *R. pomeroyi *to the thirty-six other sequenced *Roseobacter *members. For this, we performed a local tblastn to search for homologues of the thirty-nine new genes (Table 1) among the other thirty-six sequenced *Roseobacter *genomes. Table 2 compiles the eight homologous regions found that were not previously annotated as CDSs in their respective genomes. Six of these homologues belong to highly conserved genes and, as for *R. pomeroyi*, were simply missed during annotation. Interestingly, the novel identified targets, SPO_PG009 and SPO_PG020, showed a distant homologue in another *Roseobacter *strain (*R. bacterium *KLH11 and *R. bacterium *HTCC2083, respectively), meaning that these are no longer unique to *R. pomeroyi*.

**Table 2:** List of novel genes detected after extending the data obtained in *R.pomeroyi *to 36 other *Roseobacter *members

Target	Roseobacter strain	GenBank locus	5' start	3' stop	E-value
SPO_PG009	*Rhodobacterales bacterium *KLH11	DS999531.1	1860966	1860490	4e-21
SPO_PG019	*Octadecabacter antarcticus *238	DS990628.1	40225	39680	2e-27
	*Octadecabacter antarcticus *307	DS990575.1	845882	846427	5e-26
SPO_PG020	*Rhodobacterales bacterium *HTCC2083	DS995276.1	2703488	2704012	3e-26
SPO_PG024	*Phaeobacter *sp. Y4I	DS995281.1	1272857	1271454	1e-158
SPO_PG029	*Roseobacter *sp. MED193	CH902583.1	691858	690500	3e-91
	*Octadecabacter antarcticus *238	DS990628.1	672254	670707	8e-33
SPO_PG032	*Rhodobacterales bacterium *HTCC2083	DS995276.1	1621388	1621861	4e-28

The confidently detected N-terminal sequences of the 486 CDSs listed in Additional file 6 were used to check whether some of their homologues found in the other sequenced *Roseobacter *strains had been wrongly annotated. For this, a local tBLASTn analysis with the 486 protein sequences was performed for all the annotated CDSs of each of the thirty-six sequenced *Roseobacters*. A total of 9887 polypeptides sharing high similarities with these *R. pomeroyi *polypeptides were found and are listed in Additional file 7. The sequence and position of the first sixty amino acids of the alignment are also shown. In most cases, a consensus between the identified start codon of the *R. pomeroyi *protein and its respective homologue in other *Roseobacter *members was observed. Nevertheless, in 1082 cases the position of the most-N-terminal sequence annotated was not in agreement with the N-terminus of the *R. pomeroyi *protein. For all of these, we propose the correction of the annotation after manual inspection. We checked whether the new gene starts corresponded to a canonical initiation codon (ATG, GTG, or TTG) and, in the case of a sequence extension at the N-terminus, if there was evidence of sequence similarities with the *R. pomeroyi *polypeptide. The rate of erroneously identified start codons among the *Roseobacter *genomes was 11% (1082 of 9887). Of note, this value may be an underestimate because i) we discarded many doubtful corrections during manual inspection, and ii) most of the BLAST results mainly matched highly conserved genes that are generally better annotated by comparative genomics. The rate decreased to 6.8% when considering only those curated genomes (*i.e*. indicated as "complete" in the Roseobase; *D. shibae *DFL12, *R. denitrificans *OCh114, *Ruegeria *sp. TM1040 and *Jannaschia *sp. CCS1).

### Defining operonic structures with re-annotated genes

We found that some loci comprise several annotation errors. For example, Figure 2**Panel B **shows the locus where the two novel genes, SPO_PG09 and SPO_PG027, are encoded. As these two genes are equally oriented as the *rpsE *and *rpmD *genes, both encoding conserved ribosomal proteins, we checked whether an operonic structure could be identified. We purified RNA from cells where the two proteins had been detected, and amplified specific cDNA fragments by RT-PCR (Figure 2, **Panel B**). We found that the two genes are co-transcribed, as we obtained RT-PCR amplification of the SPO_PG027-SPO_PG09 region. Despite this, no amplification could be obtained between this operon and its downstream *rplO *or upstream *rpmD *genes. The latter result was expected because of a putative Rho-independent transcriptional terminator identified by the TransTerm software (Figure 2, **Panel B**).

The neighbouring genes in the operon structures of the newly annotated CDSs may infer plausible hints about the functions of the novel detected genes. This is the case for SPO_PG026, which we have shown here to be co-transcribed with the SPO_3673 gene. We have previously shown that the latter encodes an RTX-like toxin which is abundantly secreted in the culture medium [24]. RTX operons always comprise the toxin gene itself together with other genes involved in toxin activation and export [25]. SPO_PG026 could be involved in such activation or export functions. SPO_PG018 is also part of a putative RTX-like operon. We detected, by RT-PCR amplification, an operon structure comprising this gene and its flanking SPO_0490 and SPO_0491 genes. SPO_0490 shows sequence similarities with calcium-binding RTX toxins. This protein has a putative signal peptide for export as identified by the SignalP predictor software [26].

RT-PCR amplification was also performed for the SPO_PG023 and SPO_1339 couple, possibly with a linked function. SPO_1339, a signal-recognition, particle-docking protein, is located just upstream of SPO_PG023. Another operon structure detected was that of SPO_PG010 with the upstream SPO_2211-SPO_2212-SPO_2213 genes. These three genes encode the determinants of branched-chain amino acid catabolism (acyl-CoA dehydrogenase, enoyl-CoA hydratase and 3-hydroxyisobutyrate dehydrogenase). SPO_PG010 shows similarities with only a few proteins found in other *Roseobacter *members (*Oceanicola batsensis *HTCC2597, *Roseovarius *sp. HTCC2601 and *Sagittula stellata *E-37). The loci encoding these far-homologues are also located close to genes with functions related to branched-chain amino acid metabolism. However, the genomic context is not always similar to that found in *R. pomeroyi*. On the other hand, we found that SPO_PG022 is co-transcribed with its downstream gene, SPO1095, and upstream gene, SPO1094, a putative propionyl-CoA carboxylase. SPO1094 converts propionyl-CoA (a derivative product from branched-chain amino acid catabolism) to methylmalonyl-CoA (a precursor of the citric acid cycle component, succinyl-CoA). Strikingly, SPO_PG022 shares similarity with one other annotated gene, in the *Roseobacter *sp MED193, which also shows the same putative operon structure.

SPO_PG005 and SPO_PG006, shown in Figure 4, and SPO_PG016, are encoded in the close neighbourhood of the operon structures encoding essential enzymes of the central metabolic citric acid cycle in *R. pomeroyi*. Interestingly, SPO_PG006 and SPO_PG016 were shown to be co-transcribed with the genes encoding these enzymes. SPO_PG005, which is co-transcribed with the SPO_0345 gene (whose product has no assigned function), is independently transcribed from its flanking operons, *sucCD *and *sucAB *(Figure 4). SPO_PG006 belongs to the *sucCD *operon and is located between the *sucC *and *sucD *genes. Figure 4 shows the genomic context for the *sucCDAB *genes for seven other *Rhodobacteraceae *bacteria. The presence of additional CDSs in the *sucCD *operon is exclusive to the *Roseobacter *strains. Conversely, the minimal structure observed in other *Rhodobacteraceae *is the common rule amongst bacteria belonging to other clades (e.g. *Pseudomonas *or *Bacillus*). In all *Roseobacter *species that were compared, we observed a variable pattern of novel genes inserted within the *sucCD *operon and in the intergenic region between *sucAB *and *sucCD*. These novel genes are poorly conserved between the different *Roseobacter *members, as shown in Figure 4. The gene encoding SPO_PG016 is found inserted within the succinate dehydrogenase operon, *sdhCDAB*. We demonstrated its co-transcription with its flanking genes, SPO0360 and SPO0361, by a specific RT-PCR amplification. The presence of non-conserved genes included in the *sdhCDAB *operon is also common amongst *Roseobacter *members, but not in other bacteria.

## Discussion and conclusions

From the large dataset of MS/MS spectra (1 117 372) recorded for proteome samples of *R. pomeroyi *cultivated in various conditions, we identified thirty-nine newly annotated genes and nine wrongly described ORFs. We also corrected seventy-four start codons and described five sequencing errors (a base insertion in all cases) that consequently modified the characteristics of the genes encoded at these loci. Because of its environmental relevance, the *Roseobacter *clade is currently subject to intense sequencing efforts [27-31]. However, because of the large diversity of this bacterial group, there are insufficient numbers of near-related genome sequences to improve their annotations by comparative genomics alone. Here, we show the importance of proteogenomics input for a better characterization of bacterioplankton.

We noted that the number of annotation inaccuracies, in terms of structural annotation, is rather large for *R. pomeroyi *genome annotation. This is similar to previous proteogenomic reports for *Shewanella *or *Mycobacterium *bacteria that resulted in thirty-eight and twenty-nine new annotations, respectively [19,32]. In contrast, a recent proteogenomic study carried out on the enterobacterium *Yersinia pestis *identified only four novel genes [20]. As enterobacteria are the most extensively studied organisms, and numerous genomes from the *Enterobacteriaceae *family have now been sequenced and annotated, it is reasonable to consider that their genomes are amongst the best for accuracy and reliability. This is in full agreement with the proteogenomic data presented by Payne et al. [20]. Here we have shown that even highly expressed genes and operons with potentially important cellular roles were missed during the genome annotation of *R. pomeroyi*. The majority of annotation problems come from the identification of CDSs exclusive to a small number of organisms, as comparative genomics is not useful in such a case in confirming the ORF prediction. Their validation requires additional experimental evidence, such as described here. Blending data from complementary approaches, such as protein characterization by tandem mass spectrometry and transcriptomic evidence, is time consuming but results in stronger evidence for small genes. In terms of mass spectrometry, 'one-hit-wonders' are proteins identified with only one, non-redundant peptide tag. They are usually proteins with low molecular weight that are able to generate only a few tryptic peptides. Depending on the score of MS/MS spectrum assignment, these hits may be difficult to ascertain confidently and require manual validation. Gupta et al [33] proposed a method to validate one-hit-wonders using comparative proteogenomics, but this requires the recording of various MS/MS datasets on several species. Here, we used RT-PCR to detect the expression of several CDSs identified with only one peptide. In this way we obtained evidence that the locus was being expressed, giving higher confidence to the assignment. This method proved to be effective, with the addition of five novel genes to our list.

Another frequent problem encountered during genome annotation is the identification of a CDS located in two different reading frames that clearly encodes a unique, conserved protein. This can be either a real frameshift process occurring for the regulation of protein synthesis, an artefact resulting from a sequencing error, or a pseudogene that has been recently inactivated. As we identified in the present study peptides in different reading frames at the same loci (e.g. SPO_PG036 and SPO_PG037), we confirmed the production of the polypeptides encoded and discounted the existence of pseudogenes. We checked systematically the sequences of the five loci and found in all cases that the plausible frameshifts resulted from sequencing errors. The insertion of an extra nucleotide in the sequence produced a slippage of the coding region to another reading frame in the five cases. This was expected as frameshifts are rare processes of regulation and usually down-regulate the protein synthesis in bacteria, while they are frequent in Archaea or viruses [34,35]. The number of sequencing errors found in the *R. pomeroyi *genome sequence also supports the current idea of re-sequencing genomes which were established a decade ago [14]. Here we have confirmed the input of proteogenomics to indicate the specific loci that need such sequence re-evaluation which has already been highlighted by others [36].

It is worth noting that ortho-proteogenomic extension of the corrected annotations to phylogenetically-related microorganisms reinforces the interest of proteogenomic studies for poorly studied bacterial phyla. Ortho-proteogenomic analyses have, to date, been limited to only two genera, *Mycobacterium *[19] and *Yersinia *[20], and have not been extended beyond this taxonomic level. In the present work, we exploited the MS/MS data combined with comparative genomics to extend re-annotations for genomes from higher taxonomic ranks. Although all sequenced members of the *Roseobacter *clade are distantly related, they all form a robust cluster with a high rate of similarities at the 16S RNA nucleotide sequence level [37]. We have successfully extended the identified N-terminal annotation of the 486 proteins detected in *R. pomeroyi *to 9887 homologous genes in the thirty-six sequenced *Roseobacter *isolates, corresponding to nineteen distinct genera. In this way, 1082 genes that were wrongly annotated were confidently corrected. This represents 11% of the total number of ORFs considered. To highlight the importance of manual curation of genome annotations, the rate of erroneous N-terminal identifications decreased to 6.8% when considering only the four complete *Roseobacter *genomes included in this study. These error rates are probably slightly underestimated as we only considered the conserved and obvious corrections. It is important to note that the full rate of badly annotated N-terminal genes established on the well-annotated genome of *R. pomeroyi *was 12.8%. A more comprehensive annotation of the clade could only be accomplished by integrating a comparative proteogenomic analysis of various *Roseobacter *strains, as previously carried out with the genus *Shewanella *(Gupta et al 2008).

Finally, the identification of operon structures by RT-PCR has given insights of the plausible function of the novel proteins identified in the present study. Bacterial genomes are usually well structured and regulated in the form of operons. Remarkably, we found that most of the novel, proteogenomic-detected CDSs were identified in operons encoding catabolic functions for amino acid degradation, RTX-like secreted proteins or central citric cycle metabolism. Because most of the biological conditions were carried out in a peptide broth [18], this catabolism is privileged and such discovery may be advantaged. Whether the genes encoded in the close neighbourhood of genes specifying RTX-like toxins are part of the protein secretion system or associated factors is an interesting question, as such toxins can be abundantly secreted, as previously shown [24,38]. Moreover, we identified novel CDSs with no ascribable function in operons encoding essential determinants of the citric acid cycle. These novel CDSs are not at all conserved among other *Roseobacter *members, but their presence is a common topic restricted to members of this clade. Whether these genes encode proteins that enhance this central metabolic cycle in these bacteria or are opportunistic genes that specifically appear in this operon because of the advantage of their high expression is an open question. The presence of the novel proteins found in the citric acid cycle operon in *R. pomeroyi *could represent snapshots of how novel proteins with novel specific functions arise during evolution.

Systematic listing of CDSs in numerous microorganisms, with the help of proteogenomic evidence, should increase the accuracy of annotation software. As demonstrated here, proteogenomic evidences from bacteria belonging to orders that have, thus far, been poorly characterized, such as the *Roseobacter *clade, are necessary to improve genome and even metagenome annotations. Ortho-proteogenomic annotation extension to a whole bacterial clade has proven here to be highly valuable. Such extension could also be applied to metagenome data, taking into account higher constraints.

## Methods

### MS/MS data compilation

An extensive proteomic analysis was applied to *R. pomeroyi *consisting in 136 nanoLC-MS/MS runs acquired on proteomes and subproteomes from cells grown under 30 different culture conditions [18,24]. Briefly, we tried out exponential and stationary culture phases in rich and poor media, with different incubation parameters (salt concentration, temperature, microaerobiosis, pelleted cells, plate growth), and stresses (UV illumination, the presence of aromatic compounds). Cellular subproteomes were also included (cellular membrane, phosphocellulose enriched fraction and exoproteome). In addition, a novel set of 75 nanoLC-MS/MS runs were acquired from samples prepared from five cellular extracts of cells grown in exponential phase in marine broth and then subjected to UV stress, concentration (100× concentrated cells), or grown on benzoate or in presence of naphthalene, and then resolved by SDS-PAGE. Cell cultures, protein extraction, SDS-PAGE and trypsin digestion were performed as previously described [18,39]. MS/MS analysis was carried out with a LTQ-Orbitrap XL hybrid mass spectrometer (ThermoFisher) coupled to an UltiMate 3000 LC system (Dionex-LC Packings) using the same parameters as already published [23].

### MS/MS database search

A total of 1,117,372 MS/MS spectra were compiled from the 211 nanoLC-MS/MS runs. These were first tentatively assigned using an ORF database containing all the six-frame translated stop-to-stop protein sequences coded in the 4.6-kb genome of *R. pomeroyi *(NC_003911 and NC_006569, [22]). This ORF database contained 61,206 protein sequences of over 40 amino acids in size, as well as the bovine trypsin sequence and 22 of the most common human keratin contaminants. The total sequences presented 7,298,532 amino acid residues. This large database contains a large percentage of non-real protein sequences. Peak lists were generated with the MASCOT DAEMON software (version 2.2.2) from Matrix Science using the extract_msn.exe data import filter from the Xcalibur FT package (version 2.0.7) from ThermoFisher. Data import filter options were set at: 400 (minimum mass), 5000 (maximum mass), 0 (grouping tolerance), 0 (intermediate scans), and 1000 (threshold). MS/MS assignments were performed using the MASCOT search engine (version 2.3.2, Matrix Science) against the local database. Searches for tryptic peptides were performed with the following parameters: a mass tolerance of 5 ppm on the parent ion and 0.5 Da on the MS/MS, static modifications of carbamidomethylated Cys (+57.0215), and dynamic modification of oxidized Met (+15.9949). The maximum number of missed cleavage for trypsin was set at 1. All peptide matches with a score above its peptidic identity threshold set at *p *< 0.005 and rank 1 were filtered by the IRMa 1.26.1 software [40]. A total of 22,040 non-redundant peptides were found belonging to 2,550 polypeptide sequences. A more restricted database consisting of i) the 4,252 stop-to-stop ORFs corresponding to the annotated CDS [22] and ii) the additional sequences pointed by the first MASCOT search (173) was constructed. Thus, this database comprises 4,425 stop-to-stop ORFs sequences, totaling 1,584,061 amino acid residues. The whole MS/MS spectra dataset was searched against this reduced database using the same parameters. A total of 594,902 spectra were confidently assigned evidencing 22,805 non-redundant peptides (Additional file 1). Considering that a protein was validated when at least two different confident peptides were detected, 2,006 protein sequences were listed (Additional file 2). A total false positive rate of 0.8% and 0.2% for identification of peptide and polypeptide, respectively, was estimated using the reverse decoy database. In order to catalogue the most N-terminal peptides of the proteins, we performed a new MASCOT search on the reduced database for listing semi-tryptic peptides. We also searched for i) modification of translation starts that could arise because of an insertion of a methionine residue at GTG and TTG translation initiation codons, and ii) protein maturation consisting in the processing of the initiator Methionine, as described previously [23]. The resulting semi-tryptic peptides were further filtered to keep only those corresponding plausible most N-terminal peptides.

### Nucleic acid manipulation

Genomic DNA extraction of *R. pomeroyi *cells was performed using the DNeasy Blood and Tissue Kit (Qiagen) following manufacturer instructions for gram-negative bacteria. DNA PCR amplification was carried out with standard conditions using Taq DNA polymerase (Roche). All primers used for specific PCR amplification of the different targets are listed in Supplementary Additional file 3. Sequencing procedure was done on specific amplified sequence purified with the QIAquick PCR purification kit (Qiagen) and was performed systematically on at least two independent amplification products. Reaction was carried out using Dye Terminator Cycle Sequencing Quick Start kit (Beckman Coulter) and analysed on a CEQ 2000 XL DNA analysis system, as previously described [41]. RNA protect Bacteria Reagent (Qiagen) was used during cell harvesting in order to stabilize the RNA and avoid any degradation. RNA extraction was performed with the RNeasy mini kit (Qiagen) using lysozyme for bacterial lysis and RNase-free DNase (Qiagen) for DNA digestion. PCR amplification was carried out systematically to test for total DNA digestion prior to RT-PCR reaction. RNA quantification was performed with a NanoDrop ND-1000 spectrophotometer. RT-PCR was carried out using the Titan One Tube RT-PCR kit (Roche). PCR amplifications were visualised on 2% agarose gels stained with ethidium bromide.

### Genome and protein sequence analysis

Visualization of genome sequences and gene positioning was done with the Artemis browser (release 12.0,[42]). The TransTerm software [43] was used to search for Rho-independent signals for transcription termination. Local BLAST analysis was carried out using the BioEdit sequence editor v7.0.9.0 [44]. We carried out a local TBLASTN analysis searching amongst the other 36 sequenced *Roseobacters *for genes specifying similar proteins to those detected by proteogenomics in *R. pomeroyi*. These strains were: *Citriecella *sp. SE45, *Dinoroseobacter shibae *DFL 12, *Jannaschia *sp. CCS1, *Loktanella vestfoldensis *SKA53, *Maritimibacter alkaliphilus *HTCC2654, *Oceanibulbus indolifex *HEL-45, *Oceanicola batsensis *HTCC2597, *Oceanicola granulosus *HTCC2516, *Octadecabacter antarcticus *238, *Octadecabacter antarcticus *307, *Pelagibaca bermudensis *HTCC2601, *Phaeobacter gallaeciensis *2.10, *Phaeobacter gallaeciensis *BS107, *Phaeobacter *sp. Y4I, *Rhodobacteraceae *KLH11, *Rhodobacterales bacterium *HTCC2150, *Rhodobacterales bacterium *HTCC2255, *Rhodobacterales *HTCC2083, *Roseobacter denitrificans *OCh 114, *Roseobacter litoralis *Och 149, *Roseobacter *sp. AzwK-3b, *Roseobacter *sp. CCS2, *Roseobacter *sp. GAI101, *Roseobacter *sp. MED193, *Roseobacter *sp. SK209-2-6, *Roseovarius nubinhibens *ISM, *Roseovarius *sp. 217, *Roseovarius *sp. TM1035, *Ruegeria lacuscaerulensis *ITI-1157, *Ruegeria *sp. R11, *Ruegeria *sp. TM1040, *Ruegeria*Trich CH4B, *Sagittula stellata *E-37, *Sulfitobacter *sp. EE-36, *Sulfitobacter *sp. NAS-14.1 and *Thalassiobium *R2A62. The E-value cutoff was set at < 10^-20^. We also searched protein homologues in the other 36 *Roseobacter *proteomes with a local BLASTP tool (E-value < 10^-30^) in order to check specifically their most probable N-termini.

### Nucleotide and protein sequences

The novel Ruegeria pomeroyi DSS-3 nucleotide sequences reported in this paper have been deposited in the EMBL/GenBank Nucleotide Sequence Database under the accession numbers FR852579 (SPO_PG036) FR852580 (SPO_PG037), FR852581 (SPO_PG038), FR852582 (SPO_PG039), and FR852583 (SPO1905).

## Abbreviations

ORFs: Open Reading Frames; CDS: coding domain sequences; MS/MS: tandem mass spectrometry.

## Authors' contributions

JC-O designed and performed the experiments of microbiology and proteomics, and analyzed all the data. GC contributed to mRNA analysis and sequence verification. JA conceived, coordinated the study and participated in the analysis of the proteomic data. JC-O and JA wrote the manuscript. All authors read and approved the final manuscript.

## Supplementary Material

Additional file 1 **Non-redundant peptide list detected by MS/MS spectral attributions (p < 0.005)**. The excel table presents the characteristics of all the non-redundant peptides detected (22805 items) and their corresponding proteins. Click here for file

Additional file 2 **Stop-to-stop polypeptides detected by MS/MS**. The excel table presents the whole list (2547 items) of ORFs detected by MS/MS with their annotated function and sequence, as well as the number of non-redundant peptides assigned to each of these ORFs. Click here for file

Additional file 4 **List of novel genes encoded in the genome of *R. pomeroyi *detected by proteogenomics in this study**. The excel table presents the 39 new genes found in the present study and their characteristics. The corresponding ORF, the number of non-redundant peptides, the number of MS/MS spectra assigned, the plausible protein sequence, and the conditions where each product has been detected are listed for the 39 genes. Click here for file

Additional file 5 **Annotated CDS demonstrated to be longer due to the detection of tryptic peptides situated further N-ter than previously annotated**. The excel table presents the evidences for 10 annotated CDS that should be revised in terms of N-terminus. The corresponding ORF label, the number of non-redundant peptides, the new proteomic-corrected CDS sequence and the corresponding peptide evidences (peptide query, peptide sequence, peptide score) are indicated for each of these 10 CDS. Click here for file

Additional file 6 **Semi-tryptic peptides representing the most N-ter sequence of the stop-to-stop CDS**. The excel table presents the characteristics of all the semi-tryptic peptides detected which correspond to the most N-terminal sequence of the stop-to-stop ORF (22805 items) and their corresponding proteins. We distinguished the peptides starting with a methionine residue encoded by an ATG start codon (lines 5-136), those starting from the residue just after a removed methionine encoded by an ATG start codon (lines 138-463), the peptides starting with a methionine residue encoded by an GTG start codon (lines 465-471), those starting from the residue just after a removed methionine encoded by an GTG start codon (lines 473-489), and those starting from the residue just after a removed methionine encoded by an TTG start codon (lines 491-494). Click here for file

Additional file 7 **Ortho-proteogenomic based analysis extending the N-ter corrections done on *R. pomeroyi *to 36 other isolates of the *Roseobacter *clade**. The excel table presents the extension to 36 isolates for each of R. pomeroyi re-annotated genes (9887 proposals). The e-value obtained by BLAST is indicated for each homologue, as well as its N-terminus (60 first residues), the new CDS length, and its location on the corresponding genome. Click here for file

Additional file 3 **Primers used throughout the study for PCR amplifications**. The excel table presents the 37 primers used in the study for PCR amplification of specific genomic loci and their characteristics (sequence, position, names, melting temperature, targets). Click here for file

## References

[B1] Fleischmann RD Adams MD White O Clayton RA Kirkness EF Kerlavage AR Bult CJ Tomb JF Dougherty BA Merrick JM Whole-genome random sequencing and assembly of *Haemophilus influenzae *Rd Science 1995 269 5223 496 512 10.1126/science.7542800 7542800

[B2] Kyrpides NC Fifteen years of microbial genomics: meeting the challenges and fulfilling the dream Nat Biotech 2009 27 7 627 632 10.1038/nbt.1552 19587669

[B3] Armengaud J A perfect genome annotation is within reach with the proteomics and genomics alliance Curr Opin Microbiol 2009 12 292 300 10.1016/j.mib.2009.03.005 19410500

[B4] Poptsova MS Gogarten JP Using comparative genome analysis to identify problems in annotated microbial genomes Microbiol 2010 156 7 1909 1917 10.1099/mic.0.033811-0 20430813

[B5] Nielsen P Krogh A Large-scale prokaryotic gene prediction and comparison to genome annotation Bioinformatics 2005 21 24 4322 4329 10.1093/bioinformatics/bti701 16249266

[B6] Armengaud J Bland C Christie-Oleza JA Miotello G Microbial proteogenomics, gaining ground with the avalanche of genome sequences J Bacteriol Parasitol 2011 S3-001

[B7] Armengaud J Proteogenomics and systems biology: quest for the ultimate missing parts Expert Rev Proteomics 2010 7 65 77 10.1586/epr.09.104 20121477

[B8] Mou X Sun S Edwards RA Hodson RE Moran MA Bacterial carbon processing by generalist species in the coastal ocean Nature 2008 451 7179 708 711 10.1038/nature06513 18223640

[B9] Denoeud F Aury JM Da Silva C Noel B Rogier O Delledonne M Morgante M Valle G Wincker P Scarpelli C Annotating genomes with massive-scale RNA sequencing Genome Biol 2008 9 R175 10.1186/gb-2008-9-12-r175 19087247 PMC2646279

[B10] Ahrens CH Brunner E Qeli E Basler K Aebersold R Generating and navigating proteome maps using mass spectrometry Nat Rev Mol Cell Biol 2010 11 789 801 10.1038/nrm2973 20944666

[B11] Gstaiger M Aebersold R Applying mass spectrometry-based proteomics to genetics, genomics and network biology Nat Rev Genet 2009 10 9 617 627 10.1038/nrg2633 19687803

[B12] Yates JR Eng JK McCormack AL Mining genomes: correlating tandem mass spectra of modified and unmodified peptides to sequences in nucleotide databases Anal Chem 1995 67 18 3202 3210 10.1021/ac00114a016 8686885

[B13] Jaffe JD Stange-Thomann N Smith C DeCaprio D Fisher S Butler J Calvo S Elkins T FitzGerald MG Hafez N The complete genome and proteome of *Mycoplasma mobile* Genome Res 2004 14 8 1447 1461 10.1101/gr.2674004 15289470 PMC509254

[B14] de Groot A Dulermo R Ortet P Blanchard L Guerin P Fernandez B Vacherie B Dossat C Jolivet E Siguier P Alliance of proteomics and genomics to unravel the specificities of Sahara bacterium *Deinococcus deserti* PLoS Genet 2009 5 3 e1000434 10.1371/journal.pgen.1000434 19370165 PMC2669436

[B15] Zivanovic Y Armengaud J Lagorce A Leplat C Guerin P Dutertre M Anthouard V Forterre P Wincker P Confalonieri F Genome analysis and genome-wide proteomics of *Thermococcus gammatolerans*, the most radioresistant organism known amongst the Archaea Genome Biol 2009 10 6 R70 10.1186/gb-2009-10-6-r70 19558674 PMC2718504

[B16] Borchert N Dieterich C Krug K Schütz W Jung S Nordheim A Sommer RJ Macek B Proteogenomics of *Pristionchus pacificus *reveals distinct proteome structure of nematode models Genome Res 2010 20 6 837 846 10.1101/gr.103119.109 20237107 PMC2877580

[B17] Qiu Y Cho B-K Park YS Lovley D Palsson BA Zengler K Structural and operational complexity of the *Geobacter sulfurreducens *genome Genome Res 2010 20 9 1304 1311 10.1101/gr.107540.110 20592237 PMC2928509

[B18] Christie-Oleza JA Fernandez B Nogales B Bosch R Armengaud J Proteomic insights into the lifestyle of an environmentally relevant marine bacterium ISME J 2012 6 1 124 135 10.1038/ismej.2011.86 21776030 PMC3246242

[B19] Gallien Sb Perrodou E Carapito C Deshayes C Reyrat J-M Van Dorsselaer A Poch O Schaeffer C Lecompte O Ortho-proteogenomics: Multiple proteomes investigation through orthology and a new MS-based protocol Genome Res 2009 19 1 128 135 18955433 10.1101/gr.081901.108 PMC2612966

[B20] Payne S Huang S-T Pieper R A proteogenomic update to *Yersinia*: enhancing genome annotation BMC Genomics 2010 11 1 460 20687929 10.1186/1471-2164-11-460 PMC3091656

[B21] Buchan A Gonzalez JM Moran MA Overview of the marine *Roseobacter *lineage Appl Environ Microbiol 2005 71 10 5665 5677 10.1128/AEM.71.10.5665-5677.2005 16204474 PMC1265941

[B22] Moran MA Buchan A Gonzalez JM Heidelberg JF Whitman WB Kiene RP Henriksen JR King GM Belas R Fuqua C Genome sequence of *Silicibacter pomeroyi *reveals adaptations to the marine environment Nature 2004 432 7019 910 10.1038/nature03170 15602564

[B23] Baudet M Ortet P Gaillard J-C Fernandez B Guerin P Enjalbal C Subra G de Groot A Barakat M Dedieu A Proteomics-based refinement of *Deinococcus deserti *genome annotation reveals an unwonted use of non-canonical translation initiation codons Mol Cell Proteomics 2010 9 2 415 426 10.1074/mcp.M900359-MCP200 19875382 PMC2830850

[B24] Christie-Oleza JA Armengaud J In-depth analysis of exoproteomes from marine bacteria by shotgun liquid chromatography-tandem mass spectrometry: the *Ruegeria pomeroyi *DSS-3 case-study Mar Drugs 2010 8 2223 2239 10.3390/md8082223 20948905 PMC2953401

[B25] Linhartová I Bumba L Mašín J Basler M Osička R Kamanová J Procházková K Adkins I Hejnová-Holubová J Sadílková L RTX proteins: a highly diverse family secreted by a common mechanism FEMS Microbiol Rev 2010 34 1076 1112 20528947 10.1111/j.1574-6976.2010.00231.x PMC3034196

[B26] Bendtsen JD Nielsen H von Heijne G Brunak S Improved prediction of signal peptides: SignalP 3.0 J Mol Biol 2004 340 4 783 795 10.1016/j.jmb.2004.05.028 15223320

[B27] Kang I Oh HM Vergin KL Giovannoni SJ Cho JC Genome sequence of the marine alphaproteobacterium HTCC2150, assigned to the Roseobacter clade J Bacteriol 2010 192 23 6315 6316 10.1128/JB.01088-10 20889754 PMC2981202

[B28] Kang I Vergin KL Oh HM Choi A Giovannoni SJ Cho JC Genome sequence of strain HTCC2083, a novel member of the marine clade Roseobacter J Bacteriol 2011 193 1 319 320 10.1128/JB.01268-10 21036993 PMC3019945

[B29] Thrash JC Cho JC Ferriera S Johnson J Vergin KL Giovannoni SJ Genome sequences of Pelagibaca bermudensis HTCC2601T and Maritimibacter alkaliphilus HTCC2654T, the type strains of two marine Roseobacter genera J Bacteriol 2010 192 20 5552 5553 10.1128/JB.00873-10 20729358 PMC2950497

[B30] Thrash JC Cho JC Vergin KL Giovannoni SJ Genome sequences of Oceanicola granulosus HTCC2516(T) and Oceanicola batsensis HTCC2597(TDelta) J Bacteriol 2010 192 13 3549 3550 10.1128/JB.00412-10 20418400 PMC2897662

[B31] Wagner-Dobler I Ballhausen B Berger M Brinkhoff T Buchholz I Bunk B Cypionka H Daniel R Drepper T Gerdts G The complete genome sequence of the algal symbiont Dinoroseobacter shibae: a hitchhiker's guide to life in the sea ISME J 2010 4 1 61 77 10.1038/ismej.2009.94 19741735

[B32] Gupta N Tanner S Jaitly N Adkins JN Lipton M Edwards R Romine M Osterman A Bafna V Smith RD Whole proteome analysis of post-translational modifications: applications of mass-spectrometry for proteogenomic annotation Genome Res 2007 17 9 1362 1377 10.1101/gr.6427907 17690205 PMC1950905

[B33] Gupta N Benhamida J Bhargava V Goodman D Kain E Kerman I Nguyen N Ollikainen N Rodriguez J Wang J Comparative proteogenomics: Combining mass spectrometry and comparative genomics to analyze multiple genomes Genome Res 2008 18 7 1133 1142 10.1101/gr.074344.107 18426904 PMC2493402

[B34] Baranov PV Fayet O Hendrix RW Atkins JF Recoding in bacteriophages and bacterial IS elements Trends Genet 2006 22 3 174 181 10.1016/j.tig.2006.01.005 16460832

[B35] Cobucci-Ponzano B Guzzini L Benelli D Londei P Perrodou E Lecompte O Tran D Sun J Wei J Mathur EJ Functional characterization and high-throughput proteomic analysis of interrupted genes in the archaeon *Sulfolobus solfataricus* J Proteome Res 2010 9 5 2496 2507 10.1021/pr901166q 20192274

[B36] Deshayes C Perrodou E Gallien S Euphrasie D Schaeffer C Van-Dorsselaer A Poch O Lecompte O Reyrat JM Interrupted coding sequences in Mycobacterium smegmatis: authentic mutations or sequencing errors? Genome Biol 2007 8 2 R20 10.1186/gb-2007-8-2-r20 17295914 PMC1852416

[B37] Newton RJ Griffin LE Bowles KM Meile C Gifford S Givens CE Howard EC King E Oakley CA Reisch CR Genome characteristics of a generalist marine bacterial lineage ISME J 2010 4 6 784 798 10.1038/ismej.2009.150 20072162

[B38] Christie-Oleza JA Pina-Villalonga JM Bosch R Nogales B Armengaud J Comparative proteogenomics of twelve Roseobacter exoproteomes reveals different adaptive strategies amongst these marine bacteria Mol Cell Proteomics 2012 11 2 M111.013110 10.1074/mcp.M111.013110 22122883 PMC3277765

[B39] Clair G Roussi S Armengaud J Duport C Expanding the known repertoire of virulence factors produced by *Bacillus cereus *through early secretome profiling in three redox conditions Mol Cell Proteomics 2010 9 7 1486 1498 10.1074/mcp.M000027-MCP201 20368289 PMC2938089

[B40] Dupierris V Masselon C Court M Kieffer-Jaquinod S Bruley C A toolbox for validation of mass spectrometry peptides identification and generation of database: IRMa Bioinformatics 2009 25 15 1980 1981 10.1093/bioinformatics/btp301 19420053

[B41] Armengaud J Fernandez B Chaumont Vr Rollin-Genetet Fo Finet Sp Marchetti C Myllykallio H Vidaud C Pellequer J-L Gribaldo S Identification, purification, and characterization of an eukaryotic-like phosphopantetheine adenylyltransferase in the hyperthermophilic archaeon *Pyrococcus abyssi* J Biol Chem 2003 278 33 31078 31087 10.1074/jbc.M301891200 12756245

[B42] Rutherford K Parkhill J Crook J Horsnell T Rice P Rajandream M-Al Barrell B Artemis: sequence visualization and annotation Bioinformatics 2000 16 10 944 945 10.1093/bioinformatics/16.10.944 11120685

[B43] Jacobs GH Chen A Stevens SG Stockwell PA Black MA Tate WP Brown CM Transterm: a database to aid the analysis of regulatory sequences in mRNAs Nucleic Acids Res 2009 37 D72 D76 10.1093/nar/gkn763 18984623 PMC2686486

[B44] Hall TA BioEdit: a user-friendly biological sequence alignment editor and analysis program for Windows 95/98/NT Nucl Acids Symp Ser 1999 41 95 98

